# Effect of Caffeine on the Skeletal System—A Review of Experimental Studies

**DOI:** 10.3390/nu18132089

**Published:** 2026-06-26

**Authors:** Paulina Stańczak, Wiktor Krzysztofik, Wiktoria Rudolf, Kacper Grzywnowicz, Joanna Folwarczna

**Affiliations:** Department of Pharmacology, Faculty of Pharmaceutical Sciences in Sosnowiec, Medical University of Silesia, Katowice, Jagiellońska 4, 41-200 Sosnowiec, Poland; palinkastanczak@gmail.com (P.S.); wiktor123149@interia.pl (W.K.); rudolfwiktoria@gmail.com (W.R.); kacper.grzywnowicz@sum.edu.pl (K.G.)

**Keywords:** caffeine, bone metabolism, bone mineral density, osteoporosis, dietary factors, coffee consumption, bone remodeling, osteogenesis, animal studies, in vitro studies

## Abstract

Background: Caffeine is one of the most widely consumed bioactive compounds worldwide. The available data regarding its effects on bone metabolism and skeletal health remain inconsistent. The aim of this study was to review experimental studies on the effects of caffeine on the skeletal system. Methods: A literature search was conducted using PubMed to identify original experimental studies investigating the effects of caffeine on the skeletal system published up to December 2025. The reviewed studies included in vivo studies on different animal models and in vitro studies on bone-related cells. Due to data heterogeneity, a narrative analysis was performed. Results: Fifty-three studies on caffeine effects were included in the review. The findings indicate that the effects of caffeine are dose-dependent and bidirectional. Low-to-moderate doses in vivo generally exerted neutral or sometimes beneficial effects on the skeletal system, whereas higher doses were associated with impaired bone development, reduced mineralization, and increased bone loss. In estrogen-deficient animals, moderate doses showed potential protective effects, while high doses exacerbated bone loss. In vitro studies demonstrated concentration-dependent effects, with high concentrations often reducing cell viability and osteogenic activity. Conclusions: The effects of caffeine on the skeletal system are complex and context-dependent. While high exposure may adversely affect bone, low-to-moderate intake appears to be safe and may exert beneficial effects under specific conditions.

## 1. Introduction

Caffeine (1,3,7-trimethylxanthine) is a naturally occurring purine alkaloid that belongs to the methylxanthine group and is considered the most widely consumed psychoactive substance (psychostimulant) in the world [1,2,3]. Caffeine occurs in over 60 plant species, primarily in coffee (*Coffea arabica* L. and *Coffea canephora* Pierre ex A. Froehner), tea (*Camellia sinensis* (L.) Kuntze), cocoa (*Theobroma cacao* L.), cola nut (*Cola acuminata* (P. Beauv.) Schott & Endl.), guarana (*Paullinia cupana* Kunth), and yerba mate (*Ilex paraguariensis* A. St.-Hil.) [2,4,5,6]. Caffeine is widely consumed in beverages and food products such as coffee, tea, chocolate, carbonated soft drinks, and energy drinks, with coffee being the primary source of caffeine intake among adults worldwide [7,8,9]. Due to the high global consumption of coffee (and other caffeine-containing beverages), even relatively minor health effects can have significant public health implications [10].

The popularity of products containing caffeine is mainly due to their psychostimulant effects, which include increased alertness, improved concentration, and reduced fatigue [1,11]. In addition to its stimulating effects, caffeine is associated with numerous potential health benefits, including effects on the cardiovascular, nervous, and endocrine systems as well as the liver [8,12,13,14,15,16,17]. Caffeine may also exert adverse effects on the human body, including anxiety, insomnia, depression, cardiovascular symptoms such as tachycardia and hypertension, gastrointestinal disturbances, increased risk of adverse pregnancy outcomes, and toxic effects at high doses [1,7,8,18,19,20]. For many years, the effects of caffeine on the skeletal system were considered potentially adverse, based on earlier studies in humans [21,22]; more recent epidemiological studies on coffee (or other caffeine-related beverages) consumption suggest a heterogeneous effect of caffeine on bone health. However, findings from both epidemiological and clinical studies remain inconclusive, with inconsistent evidence regarding osteoporosis/fracture risk [10,23,24,25,26,27,28,29].

According to the European Food Safety Authority (EFSA), regular consumption of caffeine from all dietary sources at doses of up to 400 mg per day by healthy adults does not present any safety concerns, while habitual consumption of up to 200 mg per day during pregnancy is considered safe for the fetus [30]. For children, it is suggested that caffeine intake should not exceed 2.5 mg/kg of body weight per day [2]. However, excessive and prolonged caffeine intake, particularly at doses exceeding 400 mg per day, may lead to adverse effects such as irritability, insomnia, tachycardia, headaches, and gastrointestinal disturbances [2,3,11].

### 1.1. Mechanism of Action of Caffeine

The biological effects of caffeine are mediated by many different molecular mechanisms and are dose-dependent. The primary mechanism of action is the non-selective antagonism of adenosine receptors, particularly the A_1_ and A_2A_ subtypes, resulting from the structural similarity between caffeine and adenosine [31,32]. The blockade of these receptors modulates several neurotransmitter systems, including dopaminergic, glutamatergic, cholinergic, serotonergic and noradrenergic signaling [6,13,33,34]. At higher concentrations, caffeine also acts as a non-selective phosphodiesterase (PDE) inhibitor, leading to an increase in intracellular cyclic adenosine monophosphate (cAMP) levels [13,31,35]. Caffeine can also act as a ryanodine receptor (RyR) agonist, stimulating the release of intracellular Ca^2+^ [13], and interact with GABA_A_ receptors, modulating neuronal excitability [36,37]. In addition to its neuropharmacological effects, caffeine exhibits antioxidant, anti-inflammatory and anti-apoptotic properties, including reducing oxidative stress and modulating inflammatory mediators such as cytokines and C-reactive protein [17,38,39,40].

### 1.2. Bone and Osteoporosis

The skeletal system plays a fundamental role in maintaining the body’s structure and physiological homeostasis. Bones provide mechanical support, enable movement, protect internal organs, and serve as a reservoir for essential minerals such as calcium and phosphorus [41].

Bone tissue, which is a type of connective tissue, is metabolically very active. Throughout life, bones undergo modeling and remodeling [42]. Modeling, which determines bone growth and changes in shape, as well as the achievement of peak bone mass, occurs primarily during childhood and adolescence [43]. In adults, bone remodeling, the purpose of which is to maintain bone strength by responding to bone microdamage and to ensure calcium balance in body fluids, primarily takes place. Remodeling involves the replacement of small amounts of old bone with new bone without altering the anatomical structure through bone resorption and subsequent bone formation [42]. In adults, approximately 10% of bone undergoes remodeling per year [44]. In youth, bone formation processes predominate, while with aging, resorption processes take precedence, leading to a gradual bone loss; this process accelerates after menopause due to cessation of estrogen production [45].

Specific bone cells include osteoclasts, osteoblasts and osteocytes. The cells responsible for bone resorption are osteoclasts, which are large, multinucleated cells, deriving from hematopoietic stem cells [46]. The cells responsible for the formation of new bone (bone matrix production and its mineralization) are osteoblasts, which derive from mesenchymal stem cells. Osteocytes, into which osteoblasts differentiate, are surrounded by a mineralized bone matrix and constitute approximately 90–95% of bone cells. Bone matrix is composed of organic components, primarily type I collagen, and inorganic components, mainly hydroxyapatite, deposited on collagen fibers [42,47].

Bone remodeling is regulated by endocrine mechanisms (including parathyroid hormone, calcitonin, vitamin 1,25(OH)_2_D_3_, estrogens, glucocorticoids) and paracrine and autocrine mechanisms (mediated by cytokines, growth factors, and prostaglandins) [42,47]. A major role in the regulation of bone metabolism is played by the RANKL/RANK signaling pathway in osteoclastic lineage cells and the Wnt/β-catenin signaling pathway in osteoblastic lineage cells [48,49,50]. Bone metabolism is influenced by numerous other factors, including dietary components such as calcium and vitamin D, hormonal regulation, mechanical loads associated with physical activity, and environmental or lifestyle-related factors [51,52,53]. For lifestyle-related factors, the potential impact of substances such as caffeine on bone metabolism and bone mineral density (BMD) has also been taken into account [24,25,28,29,54].

Precise regulation of bone remodeling is essential for skeletal homeostasis, as imbalances between bone formation and resorption can lead to metabolic bone diseases such as osteoporosis [55]. Osteoporosis is a systemic disease of the skeletal system characterized by reduced bone mass, deterioration of bone microarchitecture, and increased bone fragility, leading to a higher risk of fractures, particularly of the spine, forearm and hip [56,57,58]. The prevalence of osteoporosis increases with age and differs between sexes; hormonal changes, including postmenopausal estrogen deficiency, and age-related bone loss are the most common causes of osteoporosis [56,57]. Approximately one in three women and one in five men over the age of 50 years are expected to experience osteoporotic fractures during their lifetime, underscoring the substantial global burden of this disease [59].

### 1.3. Effects of Caffeine on the Skeletal System in Humans

Studies on the effects of caffeine consumption on bone metabolism, bone mineral density, and fracture risk in humans have yielded inconsistent results. There are studies suggesting that high coffee or caffeine intake may be associated with a decrease in BMD/increase in fracture risk, particularly in older adults and postmenopausal women [24,60,61]. However, other studies have suggested that moderate coffee consumption may not adversely affect bone health and may even be beneficial [29,62,63]. Systematic reviews and meta-analyses also indicate heterogeneous and inconclusive results regarding the association between coffee consumption and fracture risk [10,23,64,65,66,67,68]. A recent analysis, based on a combination of cross-sectional research (on data from National Health and Nutrition Examination Survey (NHANES)) and Mendelian randomization methods, demonstrated that caffeine intake is inversely associated with osteoporosis risk [26]. Some studies have suggested that the potential negative effect of caffeine on bone metabolism may be attenuated by adequate calcium (milk) intake, indicating a significant role for dietary factors [60,69]. The interpretation of epidemiological study results is further complicated by the fact that, in addition to caffeine, coffee contains numerous bioactive substances, including chlorogenic acids, diterpenes, trigonelline and melanoidins, which may also influence physiological processes and health outcomes [70,71]. Moreover, the observed associations may be influenced by other dietary and lifestyle factors. Together, the results of epidemiological studies on the relationship between coffee consumption/caffeine intake and bone health in humans remain inconclusive [10,23,26,27].

In conclusion, data from human studies do not allow a clear determination of the effect of caffeine on bone health in humans. It appears that moderate caffeine consumption is probably neutral for bone, whereas high consumption may be associated with an increased risk of fracture.

Given that human studies do not allow the effects of caffeine to be separated from those of other bioactive components of food, the aim of this study was to review the available evidence from in vitro and in vivo experimental studies investigating the effects of caffeine on bone metabolism and skeletal health.

## 2. Methods

A comprehensive literature review was conducted using the PubMed database to identify experimental studies evaluating the effect of caffeine on bone metabolism and skeletal health. The review included articles published up to December 2025. The searching strategy included combinations of the following words: caffeine, 1,3,7-trimethylxanthine, coffee, tea, yerba mate, bone, bone metabolism, bone mineral density (BMD), bone mineral content (BMC), bone mechanical properties, fracture, in vivo, in vitro, osteoporosis, osteopenia, osteoblasts, osteoclasts, osteocytes, osteogenesis, bone healing, skeletal system, bone histomorphometry, bone marrow-derived mesenchymal stem cells, rat, mouse, mice, rabbit, hamster, animal, prenatal caffeine exposure, and estrogen deficiency. Additionally, a search in Google Scholar was performed. Only original in vivo and in vitro experimental studies were included. Epidemiological and clinical studies in humans, review articles, conference abstracts and studies not directly related to skeletal outcomes were excluded. Only articles published in English were considered.

Studies were selected through a two-stage selection process, comprising a review of titles and abstracts, followed by an assessment of full texts. Based on the conducted search, 87 studies were found; 26 of them concerned prenatal caffeine exposure and eventually were not included in the main analysis of this review. Due to the complexity and importance of this topic, it should be discussed in more detail separately.

The analysis included a total of 53 experimental studies concerning in vivo and in vitro caffeine effects on the skeletal system. Moreover, we present the results of eight experimental in vivo studies on the effects of natural caffeine sources (coffee and yerba mate) on the skeletal system. Relevant data were extracted from the selected studies, including study design, experimental model, caffeine dose and duration of exposure, parameters assessed, and main results. Due to the heterogeneity of the experimental models, study designs and outcome measures, a narrative analysis was performed.

Data from the studies were organized into tables and are presented in chronological order to enable a comparison of results over time. The tables summarize the key characteristics of each study, including the experimental model, caffeine dose, duration of exposure, and the outcomes. If one article concerned more than one experimental model, the results are presented in more than one table.

Caffeine doses were presented in mg/kg/day for in vivo studies and in mM or μg/mL for in vitro studies. For the purposes of analyzing dose–response relationships in in vivo studies, we divided caffeine exposure levels in rats and mice into four groups: low dose (<20 mg/kg/day), moderate dose (20–50 mg/kg/day), high dose (50–100 mg/kg/day) and very high dose (>100 mg/kg/day).

Caffeine concentrations used in in vitro studies were categorized into low/physiological (≤0.01 mM), moderate/supraphysiological (0.01–0.1 mM), high (0.1–1 mM), and very high (>1 mM) ranges, with only concentrations below 0.05 mM corresponding to plasma levels achievable in humans [17].

## 3. Results

### 3.1. Effect of Caffeine on Young Animals During the Period of Rapid Growth

The effects of caffeine on young, rapidly growing rats are presented in Table 1.

Low and moderate doses of caffeine (3–25 mg/kg/day) generally did not cause consistent changes in the skeletal system in young rats. Bone development, mineralization, bone mass or mineral metabolism in some experiments were not significantly affected [72,73], although, for example, increased collagen synthesis in the bones of pups whose mothers were fed a protein-deficient diet was demonstrated [72]. On the other hand, rats exposed to caffeine prenatally (the dams receiving 20 mg/kg in the diet) and then receiving caffeine in their diet for 92 days, sacrificed at day 388, had strongly altered bone architecture and mineralization [74].

**Table 1:** Effect of caffeine on young animals during the period of rapid growth.

Animals	Caffeine Dose and Study Conditions	Aim	Effects	Year of Publication/Reference
3-day-old Sprague Dawley rats	10 mg/kg/day i.g. to newborns every other day between days 3–13; lactating dams fed a normal or protein-deficient diet; *n* per group: 5–10	To investigate the effect of administration of caffeine after birth on the skeletal system of newborn rats; mothers fed a protein-deficient or normal diet.	Caffeine did not significantly affect body weight, bone mass, protein, calcium content, or calcium uptake. Caffeine only increased collagen synthesis in long bones in newborn rats whose mothers were fed a protein-deficient diet.	1985 [72]
4-week-old male Sprague Dawley rats	100 mg/kg/day s.c. for 4 weeks; *n* per group: 6	To investigate the effect of caffeine administration on calcium balance, blood calcium levels, and serum levels of PTH and 1,25(OH)_2_D_3_ in immature rats.	In immature rats, caffeine caused an increase in urinary and fecal calcium excretion. Concentrations of PTH and 1,25(OH)_2_D_3_ in the blood increased from day 14.	1986 [75]
Male rats	Pregnancy and lactation: maternal caffeine intake (10 mg/kg/day in the diet) until weaning (postnatal day 22); end of the experiment on day 56; *n* per group: 10–14	To investigate the effect of maternal caffeine intake during gestation and lactation on postnatal bone growth and development in rat offspring.	Caffeine exposure in offspring of mothers receiving caffeine during gestation and lactation resulted in significant reductions in femur size, weight, volume and hardness, as well as mandible weight.	1990 [76]
21-day-old male Sprague Dawley rats	12.5 or 50 or 100 mg/kg/day in drinking water for 18 days; *n* per group: 6	To investigate the effect of caffeine on endochondral bone development and mineralization using demineralized bone particles implanted subcutaneously in immature rats.	Caffeine significantly inhibited endochondral bone development, mainly by limiting the proliferation of mesenchymal cells and their differentiation into chondrocytes. Caffeine also inhibited osteoblast maturing and ALP activity, resulting in reduced bone formation.	1993 [77]
Female Sprague Dawley rats	Pregnancy and lactation: maternal dietary caffeine intake (20 mg/kg/day) Post-weaning: dietary caffeine (20 mg/kg/day) from day 22 to day 93; end of the experiment on day 388; *n* per group: 9–11	To investigate the effect of caffeine exposure during gestation, lactation, and the rapid growth period on bone structure and composition in later life in rats.	Caffeine exposure resulted in significant alterations in bone structure, including increased femoral width and periosteal bone area/total bone area and decreased cross-sectional area of the femur, and osteocyte number per bone area. It also significantly reduced Ca, P, Zn, and hydroxyproline concentrations in both the femur and mandible, as well as Mg levels in the mandible, while no significant changes were observed in body weight, femoral length, radiographic density, and Ca/P ratio.	1994 [74]
Sprague Dawley rats	Lactation: maternal dietary caffeine intake (40 mg/kg/day) Post-weaning: dietary caffeine (40 mg/kg/day) from day 22 to day 50; *n* per group: 5–10	To investigate the effect of caffeine on bone cells and early bone development after birth in rapidly growing rats.	Early exposure to caffeine caused a reduction in osteocyte density relative to the area of femoral cross-section, mitochondrial swelling in osteoblasts and osteocytes, and retarded bone structural remodeling during skeletal development.	1996 [78]
Female Sprague Dawley rats one week after weaning	3 or 25 or 100 mg/kg/day in drinking water for 6 weeks; *n* per group: 8	To investigate the effect of long-term caffeine intake at various doses on mineral balance.	Long-term caffeine consumption had no significant effect on the balance of fluoride, calcium and phosphorus or their levels in plasma, bones or enamel in rats. Only the highest dose of caffeine reduced ash content in the femur and bone mineral content in the tibia.	1999 [73]
5-week-old male Wistar rats	100 mg/kg/day in drinking water 3 times a week for 10 weeks; *n* per group: 8–11	To investigate the effect of high doses of caffeine and/or exercise on the growth of long bones, growth plate structure, BMD and trabecular parameters in growing rats.	Caffeine administration increased growth plate activity and tibia length but impaired bone quality (decreased trabecular bone volume ratio, mineralization, BMD, femoral bone mass, and calcium content). Exercise did not counteract most of the caffeine-induced changes.	2002 [79]
3-week-old male Wistar rats	0.1% or 0.2% caffeine in diet (~22 mg or 44 mg/day) for 20 weeks; *n* per group: 10	To investigate the effect of long-term dietary caffeine exposure on BMD in growing rats.	Caffeine (0.2% in diet) caused a decrease in BMD as well as a decrease in calcium content in the femur and tibia.	2011 [80]
22-day-old male Sprague Dawley rats	120 or 180 mg/kg/day i.g. for 4 weeks; *n* per group: 17	To investigate the effects of high-dose caffeine exposure during rapid growth on long bone development, bone mineral parameters, and osteogenic activity in prepubertal male rats.	High doses of caffeine significantly inhibited osteogenic activity and bone mineralization during the rapid growth phase, leading to a decrease in BMD, BMC, and a slowdown in longitudinal bone growth.	2015 [81]
22-day-old male Sprague Dawley rats	20 or 60 or 120 mg/kg/day i.g. for 10/20/30/40 days; *n* per group: 5	To investigate the effects of caffeine on longitudinal bone growth, growth plate, trabecular bone architecture and growth-related hormones in immature rats.	Caffeine significantly inhibited longitudinal bone growth in growing rats in a dose- and time-dependent manner, resulting in decreased mineralization, growth plate abnormalities, and decreased concentrations of IGF-1, estradiol, and testosterone.	2016 [82]
22-day-old male Sprague Dawley rats	120 or 180 mg/kg/day i.g. for 4 weeks; *n* per group: 5	To investigate the effects of high-dose caffeine on longitudinal bone growth, growth plate mineralization, and cell proliferation in rapidly growing rats.	Exposure to caffeine significantly inhibited longitudinal bone growth by reducing the mineralization rate and growth plate chondrocyte proliferation.	2017 [83]
21-day-old male Sprague Dawley rats	120 or 180 mg/kg/day i.g. for 4 weeks; *n* per group: 5	To investigate the effect of long-term exposure to high doses of caffeine on the growth of long bones and bone mineralization in male rats during a period of rapid growth.	High doses of caffeine significantly shortened the length and reduced the mass of long bones and body length and also reduced BMC and BMD.	2017 [84]
22-day-old female Sprague Dawley rats	120 or 180 mg/kg/day i.g. for 4 or 8 weeks; *n* per group: 20	To investigate the effect of high doses of caffeine on bone growth and growth plate histomorphometry in immature female rats.	High doses of caffeine significantly reduced the length and mass of the femur and tibia, the length of the spine, BMC and BMD (particularly after 4 weeks). Exposure to caffeine significantly increased the height of the growth plate and its PZ and HZ zones, increased the number of chondrocytes in the HZ zone, reduced trabecular bone formation parameters, increased Tb.Sp and raised serum E_2_ concentrations after 4 weeks.	2026 [85]

ALP—alkaline phosphatase. BMC—bone mineral content. BMD—bone mineral density. E_2_—estradiol. HZ—hypertrophic zone. IGF-1—insulin-like growth factor 1. i.g.—intragastrically. PTH—parathyroid hormone. PZ—proliferative zone. s.c.—subcutaneously. Tb.Sp—trabecular separation.

Moderate-to-high doses of caffeine were associated with adverse effects on skeletal development. Exposure to caffeine (100 mg/kg/day) disrupted calcium homeostasis, as evidenced by increased calcium excretion in urine and feces, changes in parathyroid hormone (PTH) and 1,25(OH)_2_D_3_ concentrations [75], and inhibited endochondral ossification at high doses (100 mg/kg/day), with more pronounced structural and mineralization defects than with lower doses [77]. Caffeine (100 mg/kg) increased long bone growth but decreased bone mineralization in young rats [79]. However, other studies reported a decreased longitudinal bone growth in caffeine-treated (60–100 mg/kg) younger rats [82]. At the tissue level, caffeine caused disruption of bone cell function [78].

High-to-very-high doses of caffeine (≥100 mg/kg/day) have been consistently associated with abnormalities in bone structural and functional parameters, including reduced BMD, BMC and mineralization [80,81,83,84,85]. These effects also included inhibition of longitudinal bone growth, abnormalities in the structure of the growth plate, and reduced osteogenic activity, particularly at very high doses of caffeine (120–180 mg/kg/day) [81,82,83,84,85], indicating a clear relationship between high caffeine exposure and negative effects on skeletal development.

### 3.2. Effect of Caffeine on Healthy Adult Animals

The effects of caffeine on the bones of healthy adult animals are presented in Table 2.

In most studies, exposure to moderate doses of caffeine (20–25 mg/kg/day) did not cause significant changes in bone structure or mineralization in healthy rats [86,87]. On the other hand, moderate doses of caffeine administered to mice (20–50 mg/kg/day) showed some unfavorable effects on the skeletal system: a reduction in the trabecular bone/bone volume ratio and a decrease in BMD (20 mg/kg/day) [88] or a deterioration in the compact bone microarchitecture and BMD with simultaneous improvement in bone mechanical properties (50 mg/kg) [89].

High-to-very-high doses of caffeine (100–180 mg/kg/day) caused an increase in calcium excretion in urine and feces and induced hormonal changes related to calcium homeostasis in rats [75] as well as a reduction in BMC in rats and hamsters [84,90].

**Table 2:** Effect of caffeine on healthy animals.

Animals	Caffeine Dose and Study Conditions	Aim	Effects	Year of Publication/Reference
12–13-month-old male Sprague Dawley rats	100 mg/kg/day s.c. for 4 weeks; *n* per group: 6	To investigate the effects of caffeine intake on changes in serum calcium, PTH, 1,25(OH)_2_D and calcium balance in adult rats.	In adult rats, caffeine administration caused an increase in calcium excretion in urine and feces. Serum calcium concentrations remained stable, although a significant increase in PTH was observed after 3 and 4 weeks, which was not accompanied by an increase in 1,25(OH)_2_D concentrations.	1986 [75]
11-week-old male Sprague Dawley rats	25 or 100 mg/kg/day in drinking water for 8 weeks; *n* per group: 8–10	To investigate the effect of long-term caffeine intake on serum markers of bone metabolism and bone histomorphometric parameters.	Caffeine administration did not cause significant changes in serum ionized calcium concentrations, PTH levels or bone histomorphometric parameters. However, high-dose caffeine intake significantly increased serum osteocalcin concentrations.	1988 [87]
3-month-old female golden hamsters	25 or 100 mg/kg/day in drinking water for 17 or 32 days; *n* per group: 7	To investigate the effect of caffeine on bone structure and BMC in hamsters.	There was no significant difference in serum calcium levels and osteocyte numbers. High-dose caffeine intake for 32 days resulted in a significant decrease in BMC.	2000 [90]
15–17-week-old female Wistar rats	20 mg/kg/day p.o. for 4 weeks; *n* per group: 10	To investigate the effect of a moderate dose of caffeine on the skeletal system of rats.	Caffeine administration did not significantly affect bone mass, mineralization, mechanical properties or histomorphometric parameters in intact rats.	2013 [86]
64-day-old male Sprague Dawley rats	120 or 180 mg/kg/day i.g. for 4 weeks; *n* per group: 5	To investigate the effect of daily high-dose caffeine intake on long bones in young adult rats.	High-dose caffeine administration reduced BMC but did not affect BMD and the length and mass of long bones in young adult rats.	2017 [84]
23-week-old female Wistar rats (sham-operated)	6 mg/kg/day in the diet for 8 weeks; *n* per group: 10	To investigate the effect of chronic caffeine intake on bone mineralization and strength in healthy rats.	Caffeine intake increased urinary calcium excretion and elevated blood bone ALP levels in rats (more data in Table 3).	2024 [91]
6-week-old C57BL/6 male mice	50 mg/kg/day i.p. for 119 days; *n* per group: 10	To investigate the effect of long-term caffeine intake on mineral distribution in bones and the structural and mechanical properties of the tibia in mice.	Caffeine administration resulted in a reduction in the mean diameter and surface area in the tibia and trabecular bone volume, as well as a decrease in calcium, phosphorus and magnesium content, but did not negatively affect bone mechanical properties (maximum load or stiffness of the tibia).	2021 [89]
8-week-old female C57BL/6J mice	20 mg/kg/day i.g. for 4 weeks; *n* per group: 7	To investigate the effect of caffeine intake on bones using micro-CT analysis.	Administration of caffeine resulted in a decline in BMD and worsening of trabecular bone: a decrease in BV/TV and Tb.N and an increase in Tb.Sp.	2021 [88]

ALP—alkaline phosphatase. BMC—bone mineral content. BMD—bone mineral density. BV/TV—bone volume/tissue volume. i.g.—intragastrically. i.p.—intraperitoneally. PTH—parathyroid hormone. p.o.—per os (orally). s.c.— subcutaneously. Tb.N— trabecular number. Tb.Sp—trabecular separation.

**Table 3:** Effect of caffeine on ovariectomized animals (experimental model of estrogen deficiency).

Animals	Caffeine Dose and Study Conditions	Aim	Effects	Year of Publication/Reference
8-month-old female Sprague Dawley rats	20 mg/kg/day in the diet for 90 days; *n* per group: 8–10	To investigate the effect of long-term caffeine intake on the mechanical properties, histology and bone minerals in aged, estrogen-deficient rats.	Long-term caffeine intake resulted in decreased mechanical parameters of the femur (yield strain significantly). Caffeine also affected the mineral composition of the bone and resulted in smaller hydroxyapatite crystallite size.	1999 [92]
Young female Sprague Dawley rats, ovariectomized on day 32	Lactation: maternal dietary caffeine intake (40 mg/kg/day) Post-weaning: dietary caffeine (40 mg/kg/day) from day 22 to day 52; *n* per group: 4	To investigate the effect of early exposure to caffeine on the mechanical strength and mineral content of the bones in young estrogen-deficient rats.	Early caffeine consumption by growing rats after ovariectomy resulted in weakened femurs, which was manifested by a significant reduction in maximum stress and elastic modulus and a reduction in calcium, magnesium, and phosphorus content.	2002 [93]
15–17-week-old female Wistar rats ovariectomized 7–8 days before the start of caffeine treatment	20 mg/kg/day p.o. for 4 weeks; *n* per group: 10	To investigate the effect of moderate caffeine intake on the skeletal system in estrogen-deficient rats.	Caffeine in rats with estrogen deficiency increased bone mineralization, improved cancellous bone structure and increased the strength of both cancellous and compact bone, partially inhibiting the development of osteoporotic changes.	2013 [86]
3-month-old female Sprague Dawley rats ovariectomized 3 months before the start of caffeine treatment	100 mg/kg/day s.c. for 1 month; *n* per group: 6	To investigate the effect of high doses of caffeine on trabecular bone loss in estrogen-deficient rats.	High doses of caffeine significantly increased trabecular bone loss in estrogen-deficient rats, causing a decrease in BV/TV and Tb.N and an increase in Tb.Sp.	2018 [94]
13-week-old female Wistar rats, ovariectomized 2 weeks before the start of caffeine treatment	9.6 or 19.2 or 38.4 mg/kg/day i.g. for 13 weeks; *n* per group: 12–14	To investigate the effect of long-term caffeine intake on the biochemical markers of bone metabolism and skeletal characteristics in estrogen-deficient rats.	Long-term caffeine intake increased serum calcium levels at all tested doses, while medium and high doses significantly reduced ALP activity and only the medium dose reduced ACP activity. Caffeine did not negatively affect femur length, BMD, biomechanical strength, or bone microarchitecture in ovariectomized rats.	2019 [95]
23-week-old female Wistar rats ovariectomized 3 weeks before the start of caffeine treatment	6 mg/kg/day in the diet for 8 weeks; *n* per group: 10	To investigate the effect of long-term caffeine intake on bone health in ovariectomized rats.	Caffeine-treated rats (ovariectomized and sham-operated combined) had decreased bone calcium content in the tibia, decreased strength of the femur and increased urinary calcium excretion in relation to the placebo treated rats.	2024 [91]
C57BL/6 8-week-old female mice ovariectomized 1 week before the start of caffeine treatment	27.72 or 55.44 or 110.88 mg/kg/day i.g. for 12 weeks; *n* per group: 12	To investigate the effect of long-term intake of various doses of caffeine on bone metabolism and microarchitecture in ovariectomized mice.	Low and medium doses of caffeine showed a protective effect against OVX-induced bone loss by increasing femoral bone mass, lowering the ACP/ALP ratio, and reducing the number of TRAP-positive osteoclasts. The medium dose showed the strongest effect, significantly improving bone microstructural parameters (BMD, BV/TV, Tb.N, BS/TV) and reducing Tb.Sp. Although the high dose also improved several biochemical markers (↑E_2_, ↑ALP, ↓ACP), its protective effects were weaker and associated with a higher number of TRAP-positive cells than the medium dose.	2024 [96]

ACP—acid phosphatase. ALP—alkaline phosphatase. BMC—bone mineral content. BMD—bone mineral density. BV/TV—bone volume/tissue volume. BS/TV—bone surface/tissue volume. E_2_—estradiol. i.g.—intragastrically. OVX—ovariectomized. PTH—parathyroid hormone. p.o.—per os (orally). s.c.—subcutaneously. Tb.N—trabecular number. Tb.Sp—trabecular separation. TRAP—tartrate-resistant acid phosphatase.

### 3.3. Effect of Caffeine on Estrogen-Deficient Animals

The effect of caffeine on bone metabolism and the development of osteoporotic changes induced by estrogen deficiency was investigated in bilaterally ovariectomized (OVX) animals. The results are presented in Table 3.

It was observed that long-term administration of caffeine to OVX rats in moderate doses (20–40 mg/kg/day) slightly impaired the mechanical properties of the femur and affected bone mineral composition [92,93]. High doses of caffeine (100 mg/kg/day) significantly increased trabecular bone loss in rats, resulting in reduced bone volume/tissue volume (BV/TV) and trabecular number (Tb.N) and increased trabecular separation (Tb.Sp) [94]. Moreover, long-term administration of low-dose caffeine (6 mg/kg/day) caused a decrease in calcium content in the tibia and an increase in urinary calcium excretion [91].

However, our study [86] showed a slight but statistically significant beneficial effect of caffeine administered at a moderate dose (20 mg/kg/day), as evidenced by increased bone mineralization, improved trabecular bone structure and enhanced bone strength. This was partially reflected in another study [95], in which caffeine administered at low-to-moderate doses (9.6–38.4 mg/kg/day) altered the biochemical markers of bone metabolism (decreasing alkaline phosphatase (ALP)) without affecting the femoral length, BMD, bone microarchitecture or biomechanical strength. Similar to our results, favorable effects were observed in a recent study on mice with estrogen deficiency, where caffeine (27.72–110.88 mg/kg/day) had a protective effect against bone loss, increasing femoral mass and improving microstructural parameters, with the strongest effect observed at an intermediate dose [96].

### 3.4. Effect of Caffeine on Bones in Different Experimental Models In Vivo

The effects of caffeine on the skeletal system have been investigated in various other experimental models, including diabetes, a high-fat diet and pregnancy (Table 4).

In our study [98] on rats with experimental type 1 diabetes induced by streptozotocin and experimental type 2 diabetes induced by streptozotocin and nicotinamide, caffeine administered at a moderate dose (20 mg/kg/day) did not affect the bone changes observed in animals with metabolic disorders induced by diabetes. However, an experiment conducted on mice fed a high-fat diet showed that long-term administration of a moderate dose of caffeine (50 mg/kg/day) improved the mechanical properties of bone (the bending strength and tibia stiffness) while increasing mineral loss and impairing the structure of compact bone in the tibial shaft [89].

Very high doses of caffeine (100–120 mg/kg/day) administered during pregnancy led to a reduction in pelvic bone mineral density and a deterioration in bone mechanical properties [97] as well as a decrease in the femur length and changes in bone turnover markers and strength in pregnant female rats [99].

### 3.5. Effect of Caffeine on Dental Health (Various Models)

The effects of caffeine on dental health were studied in rats with periodontitis, periapical inflammation, healing following tooth extraction, and orthodontic tooth movement. The results are presented in Table 5.

In periodontitis models, high doses of caffeine (100 mg/kg/day) increased periodontal tissue damage and alveolar bone loss [100], reduced the trabecular bone area and increased the receptor activator of the nuclear factor kappa Β ligand (RANKL)/osteoprotegerin (OPG) ratio, indicating increased osteoclastogenic activity [101]. The high caffeine intake (100 mg/kg/day) in periapical periodontitis increased the inflammatory response, osteoclast activity and bone resorption, as indicated by the increased expression of RANKL, TRAP and interleukin 1β (IL-1β) [102].

Following tooth extraction, the administration of caffeine at a moderate dose (30 mg/kg/day) reduced the formation of new bone tissue and delayed the maturation of trabecular bone [103]. In orthodontic tooth movement models, caffeine at moderate-to-high doses (25–75 mg/kg/day) increased orthodontic tooth movement, the number of osteoclasts and markers of bone resorption whilst reducing the BV/TV ratio [104,105].

It was also demonstrated that a low dose of caffeine (10 mg/kg/day) attenuated bone loss, increased the BV/TV ratio and reduced Tb.Sp in a model of ethanol-induced alveolar bone damage [106].

### 3.6. Effect of Caffeine on Bone Healing

Experimental studies have also examined the effect of caffeine on bone regeneration in bone defects and on implant osseointegration (Table 6).

High doses of caffeine (100 mg/kg/day) adversely affected early bone healing. Chronic caffeine administration reduced the volume and organization of newly formed bone within the bone defect without affecting bone density [107]. Moreover, caffeine increased the number of TRAP-positive cells and the RANKL/OPG ratio in bone defects, indicating increased osteoclastogenic activity. In the same experiment conducted on estrogen-deficient rats, a high dose of caffeine (100 mg/kg/day) increased the RANKL/OPG ratio and reduced the expression of osteogenic genes (BMP-2, BMP-7) without affecting the trabecular bone area or bone healing [108].

Caffeine at a lower dose (300 mg/L in drinking water) in an implant osseointegration model significantly increased implant stability and mineralized bone volume within implant threads, indicating enhanced long-term osseointegration [109].

### 3.7. Effect of Natural Caffeine Sources (Coffee and Yerba Mate) in Experimental In Vivo Studies

The results of experimental studies investigating the effects of complex caffeine sources, such as coffee, coffee infusions, yerba mate, and coffee bean extracts, on bone metabolism and regeneration are presented in Table 7.

Long-term coffee consumption (added to the diet) did not affect bone turnover markers, including serum osteocalcin, or the number of osteoclasts in rats [110]. Similarly, yerba mate infusion did not negatively affect bone parameters and partially attenuated trabecular bone loss induced by calcium deficiency [113]. Yerba mate instant powder was reported to mitigate bone deterioration in old rats [115] and to increase bone area and osteocyte density after tooth extraction in rats [114].

However, prenatal and chronic coffee exposure was associated with disturbances in calcium homeostasis, including increased plasma and urinary calcium levels, decreased bone calcium content, reduced BMD and bone volume, and delayed alveolar bone repair after tooth extraction in rats [111]. Long-term coffee infusion consumption also decreased new bone formation and osseus integration in young rats [112]. On the other hand, coffee bean extract rinsed into the periodontal pocket enhanced alveolar bone regeneration by increasing osteoblast numbers, decreasing osteoclast numbers, and increasing BMP-2 expression in rats with periodontitis [116].

### 3.8. Effect of Caffeine on Osteoblasts and Osteoblast-like Cells In Vitro

In vitro studies conducted on osteoblasts and osteoblast-like cells (primary calvarial osteoblasts, MC3T3-E1, UMR106-01, SaOS-2, U2-OS, MG-63, and hFOB 1.19 cell lines) showed that the effects of caffeine on cell metabolism and function are strongly dependent on the concentration and cellular model (Table 8).

High concentrations of caffeine (0.2–10 mM) reduced osteoblast viability and activity [118,120,122], decreased vitamin D receptor (VDR) protein expression and ALP activity [121], and impaired extracellular matrix formation and mineralization [117,120]. Moreover, caffeine was reported to induce oxidative stress and apoptosis [122]. Caffeine also increased RANKL expression and reduced OPG expression (0.001–1 mM) [80].

However, other studies demonstrated different or even opposite effects depending on experimental conditions. Caffeine at 0.5 mM enhanced glucocorticoid receptor-mediated gene expression via cAMP-dependent mechanisms [119]. Low concentrations of caffeine (0.003–0.017 mM) increased cell viability in MC3T3-E1 cells [124], while moderate concentrations (12.5 μg/mL; 0.064 mM) stimulated osteogenic markers such as ALP activity and Runx2 expression [96]. Moreover, prenatal exposure to caffeine (50 mg/kg/day) was associated with increased osteogenic potential of osteoblasts, including enhanced ALP activity, collagen synthesis, and mineralization [123].

### 3.9. Effect of Caffeine on Mesenchymal Stem/Stromal Cells In Vitro

The effects of caffeine on mesenchymal stem/stromal stem cells (bone marrow-derived mesenchymal stem/stromal cells, adipose-derived stem cells, dental pulp stem cells) have been investigated in different experimental conditions (Table 9).

Caffeine at high concentrations (0.1–1.0 mM) reduced cell viability and proliferation, increased apoptosis in bone marrow-derived mesenchymal stem cells (BMSCs) [88,125], and inhibited osteogenic differentiation [88,94,125]. Also, administration of caffeine during pregnancy and lactation inhibited the osteogenic differentiation of mesenchymal stem cells derived from the rat offspring [127]. Caffeine also promoted adipogenic differentiation of BMSCs, as evidenced by increased lipid accumulation and expression of adipogenic markers [88]. In dental pulp stem cells (DPSCs), caffeine at low concentrations did not affect cell viability or proliferation, but a concentration of 8.03 µM increased adipogenic differentiation [128].

However, bidirectional effects were also observed. At a concentration of 0.1 mM, caffeine enhanced osteogenic differentiation and increased the expression of osteogenic markers such as RUNX2, OPG, and SIRT1 in adipose-derived stem cells (ADSCs), whereas higher concentrations (0.3–1 mM) resulted in inhibitory effects [126].

Furthermore, yerba mate extract (unspecified caffeine concentration) exhibited dose-dependent effects, with lower concentrations enhancing osteogenic differentiation and higher concentrations reducing viability, mineralization, and ALP activity in BMSCs [129].

### 3.10. Effect of Caffeine on Osteoclastogenesis In Vitro

In vitro studies on osteoclast precursor cells indicate that the effects of caffeine on osteoclastogenesis are dependent on the concentration and cell culture model (Table 10).

Most studies reported on increased osteoclastogenesis induced by caffeine. Caffeine at low concentrations (≤0.01 mM) enhanced osteoclast differentiation and resorptive activity, as demonstrated by an increased number of TRAP-positive multinucleated cells and increased resorption in bone marrow cell culture [80]. Caffeine at low-to-moderate concentrations also strongly increased osteoclastogenesis in RAW264.7 cells [48,130], while higher concentrations inhibited it [130]. On the other hand, it was reported that low concentrations of caffeine may inhibit osteoclastogenesis in RAW264.7 cell culture, while the highest concentration studied increased the number of TRAP-positive multinucleated osteoclasts [96].

### 3.11. Effect of Caffeine on Other Bone-Related Models In Vitro

The effects of caffeine have also been investigated in in vitro models in which calvarial bone, periodontal ligament cells, growth plate chondrocytes, and osteocyte-like cells were cultured (Table 11).

In calvarial bone in vitro culture, caffeine at various concentrations (0.026–2.6 mM) did not significantly affect calcium release into the culture medium or induce morphological changes in bone tissue [131]. However, under different experimental conditions, caffeine at high concentrations (0.3–1 mM) increased the release of ^45^Ca, indicating stimulation of bone resorption [132], while at low-to-moderate concentrations (0.005–0.1 mM), it increased COX-2 protein expression and PGE_2_ production [80].

Caffeine at low concentrations (0.01 mM) increased RANKL expression, COX-2 expression, and PGE_2_ production in periodontal ligament cells and enhanced the formation of TRAP-positive osteoclasts in co-culture systems [104].

Moreover, caffeine at high concentrations (0.1–1 mM) reduced the proliferation and chondrogenic differentiation of growth plate chondrocytes as well as extracellular matrix production and mineralization [83]. In contrast, in osteocyte-like cells, caffeine at high concentrations (approximately 0.05–0.25 mM) increased cell viability [124].

### 3.12. Effect of Caffeine on Fetuses and Offspring Skeletal Systems

Prenatal caffeine exposure has been investigated in numerous experimental studies. As stated before, the complexity and importance of the problem require separate, detailed analysis. Taken together, available evidence indicates that prenatal exposure to very high doses of caffeine (most commonly ≥100 mg/kg/day) is associated with adverse effects on fetal skeletal development as well as persistent negative outcomes observed in offspring [53,133,134,135,136,137,138,139,140,141,142,143,144,145,146,147,148,149]. Low-to-moderate doses of caffeine generally produced less pronounced detrimental effects on fetal bone development [150,151,152,153,154,155,156,157].

## 4. Discussion

Despite advances in the pharmacotherapy of skeletal disorders, osteoporosis management requires an individualized approach, as no single treatment is universally effective in all conditions [56,57,158]. Increasing attention has therefore focused on modifiable environmental and dietary factors. Among these, caffeine and caffeine-containing products have been investigated as potential modulators of bone metabolism.

The present review demonstrates that the effects of caffeine on the skeletal system are complex and strongly dependent on the dose, experimental model, and pathophysiological context. The results of the analyzed experimental studies consistently demonstrated that caffeine at high doses exerted adverse skeletal outcomes (for example, [75,77,81,84]); however low-to-moderate caffeine doses exerted generally either neutral or favorable effects [72,73,86].

Importantly, the interpretation of experimental doses requires consideration of interspecies differences. In humans, caffeine intake is typically classified as low (<200 mg/day), moderate (200–400 mg/day), and high (>400 mg/day) [34], which, assuming an average body mass of 65 kg, corresponds to approximately <3.1 mg/kg/day, 3.1–6.2 mg/kg/day and >6.2 mg/kg/day, respectively.

These doses correspond approximately to rat doses of <19, 19–38, and >38 mg/kg/day, respectively, and to even bigger mouse doses: <38 mg/kg/day, 38–76 mg/kg/day and >76 mg/kg/day [159]. The estimated lethal dose (LD_50_) of caffeine in male rats is 367 mg/kg *p.o.* [160]. Many studies included in this review used doses within or moderately above the translational range. Caffeine was administered using various routes, including in the diet or in drinking water, by oral gavage/intragastric, and by intraperitoneal or subcutaneous injection, which may influence its pharmacokinetics and biological effects. For example, intragastrical administration of caffeine by oral gavage once or twice daily seems to better correspond to coffee drinking in humans than oral administration in the diet or drinking water. As far as other routes of administration are concerned, intraperitoneal administration of small molecules usually results in faster and more complete absorption than oral or subcutaneous routes [161].

An important factor contributing to the diversity of the results analyzed is the duration of caffeine exposure in relation to the length of life and developmental stage of the experimental animals. In rodents, biological maturation occurs much more rapidly than in humans; consequently, even relatively short experimental periods may correspond to long-term exposure on a human scale. On average, one day in rat life corresponds approximately to 26.7 days of human life [162], whilst one day in mouse life corresponds to approximately 40 days of human life [163]. Consequently, experiments on rats lasting 4 weeks correspond approximately to more than 2 years of human life, whilst a 12-week exposure to a substance may reflect more than 6 years of chronic consumption.

This issue appears to be particularly significant in studies conducted on rapidly growing animals. In most experiments showing adverse effects on the skeletal system, caffeine was administered over a prolonged period, beginning shortly after weaning or during periods of rapid skeletal growth [81,82,83,84,85]. High doses of caffeine administered over 4–8 weeks impaired bone longitudinal growth and mineralization [81,83,84,85]. Considering the rapid maturation of rodents, these exposure periods may correspond to years of caffeine consumption during childhood or adolescence in humans. Similarly, the 20-week exposure in growing rats reported in [80] may reflect chronic consumption over a significant portion of skeletal maturation.

Caffeine, in addition to its widespread dietary consumption, is also a Food and Drug Administration (FDA)-approved medicinal product (as caffeine citrate), used primarily in the treatment of apnea of prematurity. Clinical studies in preterm infants have demonstrated an association between caffeine therapy and an increased risk of osteopenia of prematurity as well as reduced bone mineral content [164,165,166], indicating that the skeletal effects of caffeine may be highly dependent on the developmental and pathophysiological context.

In experimental models of estrogen deficiency, which mimic postmenopausal osteoporosis, the effects of caffeine appear to depend on dose and experimental conditions. High doses were associated with the deterioration of bone mechanical properties and increased trabecular bone loss [94]. Administration of caffeine in the diet (20 and 40 mg/kg) led to a slight worsening of bone properties of aged and young ovariectomized rats [92,93]. However, the majority of more recent studies do not confirm bone damage induced by caffeine at moderate doses. Only one study reported the deleterious effect of low-dose (6 mg/kg in the diet) caffeine [91]. Other studies, using more advanced methods, demonstrated no bone damage [95] or favorable effects [86,96]. These results suggest that, in estrogen deficiency, moderate caffeine intake may counteract bone deterioration rather than exacerbate it. We have demonstrated that moderate caffeine intake may exert protective effects in ovariectomized rats; caffeine at 20 mg/kg/day (bolus *p.o.* doses) improved bone mineralization, trabecular bone structure, and mechanical strength, partially inhibiting the development of osteoporotic changes in ovariectomized rats [86]. These findings were consistent with the results of a recent study on ovariectomized mice [96].

The cellular mechanisms underlying these effects are not fully elucidated. Caffeine acts as a non-selective antagonist of adenosine receptors (A_1_, A_2A_, A_2B_, and A_3_) [31,167]. Adenosine is involved in the regulation of bone remodeling processes, with its receptors expressed in both osteoblasts and osteoclasts [168,169,170]. Experimental data indicate that adenosine receptor subtypes may exert different effects on bone metabolism; A_1_ receptor signaling is associated with osteoclast-mediated bone resorption, whereas A_2B_ receptor activation promotes osteoblast differentiation [171,172,173]. However, the extent to which these mechanisms contribute to the skeletal effects of caffeine in vivo remains uncertain and likely depends on dose and biological context.

The results of in vitro studies further support the importance of the concentrations of caffeine investigated. Physiological plasma concentrations of caffeine in humans typically reach micromolar levels (approximately up to 50 µM) [17], whereas many in vitro experiments used concentrations in the millimolar range. This discrepancy limits the direct translational value of some in vitro findings, particularly those demonstrating reduced cell viability, impaired mineralization, or induction of apoptosis at high concentrations.

The observed effects of caffeine on osteoblasts, mesenchymal stem cells, and osteoclast precursors consistently indicate that caffeine modulates the proliferation, differentiation, and activity of bone cells in a concentration-dependent manner [48,80,88,94,96,117,118,119,120,121,122,123,125,126,127,128,129,130]. High concentrations tend to inhibit osteoblast function, whereas lower concentrations may enhance osteogenic differentiation or exert neutral effects [80,96,117,118,119,120,121,122,123]. The effects of caffeine on osteoclastogenesis are inconsistent [48,80,96,130].

Another important aspect concerns the differences between the ways by which caffeine is consumed. In human studies, caffeine intake is usually estimated based on consumption of beverages such as coffee or tea, whereas in experimental models, caffeine is administered as a pure compound, usually at precisely controlled doses. Natural sources of caffeine, including coffee and yerba mate, contain numerous bioactive compounds such as polyphenols and diterpenes, which may independently influence bone metabolism [110,113]. For example, we have demonstrated the unfavorable effect of trigonelline, an alkaloid present in coffee in considerable amounts, on the skeletal system in ovariectomized rats [174]. Therefore, the effects observed in studies using these products cannot be attributed solely to caffeine.

It should be taken into account that not only caffeine itself but also its metabolites may exert their effects on bone tissue. In both rats and humans, caffeine is metabolized by CYP1A2, and the main metabolites of caffeine are theophylline, theobromine and paraxanthine. In rats, their amounts are similar, whereas in humans, paraxanthine is a primary metabolite [175]. So far, it has been reported that theobromine administered at a low dose of 10 mg/kg to pregnant and lactating rats as well as later to the offspring accelerated skeletal development [176], whereas chronic administration of theophylline (50 and 100 mg/kg) induced severe dose and time-dependent osteopenia in adult male rats [177]. These results are consistent with the results of caffeine studies reviewed here (no data on the bone effects of paraxanthine were found).

Moreover, individual variability in response to caffeine should be considered. Genetic factors, particularly polymorphisms in the CYP1A2 gene, significantly influence caffeine metabolism, with interindividual differences in clearance reaching up to 40-fold [178]. These differences may also affect the relationship between caffeine intake and bone mineral density, as suggested by genotype-dependent associations between coffee consumption and bone health [179].

**Figure 1:**
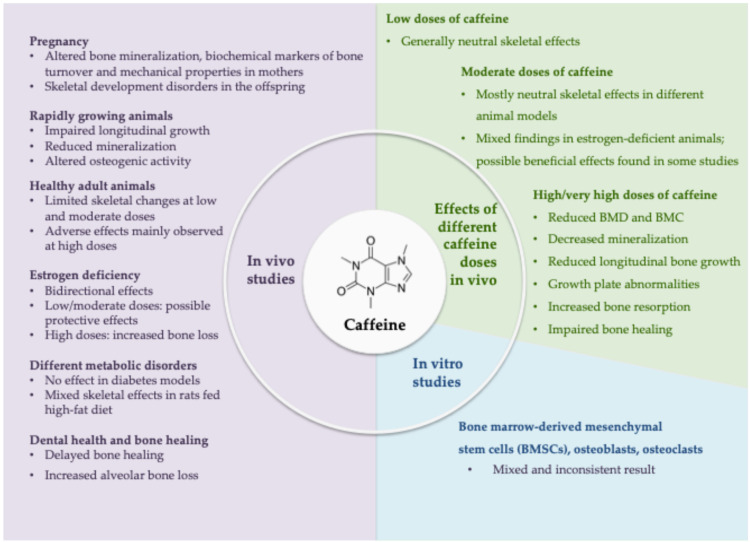
Summary of the results of experimental studies on caffeine effects on the skeletal system.

Taken together, the available evidence suggests that the effects of caffeine on the skeletal system cannot be generalized without considering dose, experimental model, and physiological context. The results of the review are summarized in Figure 1. While high doses of caffeine may exert detrimental effects on bone development and remodeling, low-to-moderate intake appears to be safe and may even exert beneficial effects on the skeletal system under certain conditions, particularly in relation to estrogen deficiency. This is consistent with growing evidence on the health benefits of moderate coffee drinking. However, further studies are required to fully recognize the mechanisms underlying coffee effects on the skeletal system and to establish the relevance of the experimental findings to human bone health.

The data presented in the review support the notion that moderate caffeine intake in adults within commonly consumed dietary ranges is unlikely to adversely affect bone health in healthy individuals. However, caution may be warranted in specific populations, such as pregnant women, children, adolescents, or individuals with low calcium intake.

## Figures and Tables

**Table 4:** Effect of caffeine on bones in different experimental models in vivo.

Animals	Caffeine Dose and Study Conditions	Aim	Effects	Year of Publication/Reference
Pregnant female Wistar rats	30 mg/day i.g. for 14 days; *n* per group: 8	To investigate the effect of caffeine intake on bones in pregnant rats.	Caffeine administration during pregnancy resulted in decreased pelvic bone density, bone stiffness, and mineral phase content as well as increased femoral shaft deformation under load in pregnant rats.	2011 [97]
3-month-old female Wistar rats with streptozotocin-induced diabetes	20 mg/kg/day p.o. for 4 weeks; *n* per group: 8–10	To investigate the effects of moderate-dose caffeine on the skeletal system of rats in experimental type 1 diabetes.	Caffeine did not affect the strong changes in bones of rats with streptozotocin-induced type 1 diabetes.	2017 [98]
3-month-old female Wistar rats with nicotinamide-streptozotocin-induced diabetes	20 mg/kg/day p.o. for 4 weeks; *n* per group: 10	To investigate the effects of moderate-dose caffeine on the skeletal system of rats in experimental type 2 diabetes.	Caffeine did not affect the slight changes in bones of rats with nicotinamide/streptozotocin-induced type 2 diabetes.	2017 [98]
6-week-old C57BL/6 male mice fed a high-fat diet	50 mg/kg/day i.p. for 119 days; *n* per group: 10	To investigate the effects of long-term caffeine intake on bone mineral balance, structure, and biomechanical properties of the tibia in mice fed a high-fat diet.	Caffeine significantly improved bone mechanical properties (bending strength and stiffness of the tibia) while increasing mineral loss in mice fed a high-fat diet. Caffeine worsened the structure of compact bone of the tibial diaphysis but increased trabeculae width in cancellous bone of the tibia.	2021 [89]
10-week-old pregnant female Wistar rats	120 mg/kg/day suspended in Tween 80 and sterile distilled water (at 10 °C or 25 °C or 45 °C) i.g. for 14 days; *n* per group: 25	To investigate the effect of caffeine intake at varied temperatures on bone characteristics in pregnant female rats.	Caffeine intake by pregnant rats, particularly when administered at 10 °C, resulted in reduced femoral length and decreased mechanical strength of bone, increasing susceptibility to fracture. Additionally, caffeine altered the biochemical markers of bone formation and turnover, indicating disturbances in skeletal metabolism.	2021 [99]

i.g.—intragastrically. i.p.—intraperitoneally. p.o.—per os (orally).

**Table 5:** Effect of caffeine on dental health (various models).

Animals	Caffeine Dose and Study Conditions	Aim	Effects	Year of Publication/Reference
90-day-old male Wistar rats with ligature-induced periodontitis	100 mg/kg/day in drinking water for 56 days; *n* per group: 10–12	To investigate the effect of long-term high-dose caffeine intake on ligature-induced periodontitis in rats.	Caffeine administration significantly increased periodontal tissue damage in ligated teeth as well as the extent of alveolar bone loss. These results suggest that long-term administration of high doses of caffeine may increase ligation-induced periodontitis in rats.	2008 [100]
90-day-old ovariectomized Wistar rats	100 mg/kg/day in drinking water for 65 days; *n* per group: 15	To investigate the effect of caffeine on ligature-induced alveolar bone loss, the trabecular bone area and bone healing after tooth extraction in ovariectomized rats.	In OVX rats, caffeine administration resulted in a significantly higher RANKL/OPG^+^ cell ratio in the alveolar bone around non-ligated teeth, while no significant differences were observed in bone loss, the trabecular bone area, bone healing, TRAP-positive cells, or the RANKL/OPG^+^ cell ratio around ligated teeth.	2013 [101]
90-day-old female Wistar rats (sham-operated)	100 mg/kg/day in drinking water for 65 days; *n* per group: 15	To investigate the effect of caffeine on ligature-induced alveolar bone loss, the trabecular bone area and bone healing after tooth extraction in non-ovariectomized rats.	Chronic caffeine administration significantly increased ligature-induced alveolar bone loss and significantly reduced the trabecular bone area. Caffeine also significantly impaired early alveolar bone healing after tooth extraction. Additionally, caffeine intake significantly increased the RANKL/OPG^+^ cell ratio in the alveolar bone around both ligated and unligated teeth, indicating enhanced osteoclastogenic activity.	2013 [101]
Male Wistar rats with experimental periapical periodontitis	100 mg/kg/day i.g. for 45 days; *n* per group: 8	To investigate the effect of excessive caffeine intake on periapical periodontitis in rats.	High caffeine intake significantly increased the inflammatory response and osteoclast activity in periapical periodontitis, indicating enhanced periapical bone resorption, as demonstrated by the larger lesion volume and significantly higher expression of RANKL, TRAP, and IL-1β.	2021 [102]
Male Wistar rats (250–300 g)	30 mg/kg/day i.p. for 30 days; *n* per group: 5	To investigate the effect of daily caffeine intake on the healing process of the alveolar bone after tooth extraction in rats (after 7, 14 and 21 days).	Caffeine significantly reduced new bone formation and delayed the maturation of trabecular bone in the extraction socket.	2015 [103]
9-week-old male Wistar rats	25 mg of caffeine/kg/day i.g. for 3 weeks (as coffee); *n* per group: 15	To investigate the effect of daily caffeine intake on orthodontic movement in rats.	Caffeine significantly increased orthodontic tooth movement and was correlated with higher numbers of TRAP-positive osteoclasts and RANKL expression on the compression side, without affecting alveolar BMD or bone volume fraction.	2016 [104]
Male Wistar rats (200–250 g)	25, 50, or 75 mg/kg/day i.p., every second day for 21 days; *n* per group: 7	To investigate the effect of caffeine on orthodontic tooth movement and histomorphometric markers of bone resorption in rats.	Caffeine administration significantly increased orthodontic tooth movement and the number of osteoclasts, blood vessels, and Howship’s lacunae while decreasing the BV/TV ratio. However, it did not significantly affect tooth root resorption.	2022 [105]
35-day-old female Wistar rats	10 mg/kg/day p.o. for 21 days; *n* per group: 5	To investigate the effect of daily low-dose caffeine intake on ethanol-induced alveolar bone damage in young female rats.	Daily low-dose caffeine intake significantly attenuated binge-like ethanol-induced alveolar bone deterioration by increasing the bone volume fraction and reducing Tb.Sp. Caffeine also significantly reduced ethanol-related vertical alveolar bone loss.	2020 [106]

BMD—bone mineral density. BV/TV—bone volume/tissue volume. IL-1β—interleukin 1-β. i.g.—intragastrically. i.p.—intraperitoneally. OPG—osteoprotegerin. OVX—ovariectomized. p.o.—per os (orally). RANKL—receptor activator of nuclear factor kappa-Β ligand. Tb.Sp—trabecular separation. TRAP—tartrate-resistant acid phosphatase.

**Table 6:** Effect of caffeine on bone healing and implant integration.

Animals	Caffeine Dose and Study Conditions	Aim	Effects	Year of Publication/Reference
90-day-old male Wistar rats	100 mg/kg/day in drinking water for 56 days; *n* per group: 12	To investigate the effect of long-term daily intake of high doses of caffeine on bone density and on early stages of bone healing in rats.	Chronic high-dose caffeine intake significantly disturbed early bone healing by reducing the amount and organization of newly formed bone within the defect but did not cause measurable changes in bone density in rats.	2009 [107]
90-day-old female Wistar rats; ovariectomy 2 weeks after start of caffeine treatment	100 mg/kg/day in drinking water for 65 days; *n* per group: 15	To investigate the effect of long-term caffeine intake on trabecular bone structure and the early stages of bone loss healing in ovariectomized rats.	Caffeine intake significantly increased the RANKL/OPG ratio in the femur but did not affect bone healing or the trabecular bone area. However, caffeine reduced the expression of BMP-2, BMP-7, and CITED-2 genes.	2014 [108]
90-day-old female Wistar rats (sham-operated)	100 mg/kg/day in drinking water for 65 days; *n* per group: 15	To investigate the effect of long-term caffeine intake on early bone healing and trabecular bone morphology in rats.	Caffeine worsened early bone healing and increased the number of TRAP-positive cells in the femur and the RANKL/OPG ratio in bone defects without significantly affecting the trabecular bone area or osteogenic gene expression.	2014 [108]
3-month-old female Wistar rats	300 mg/L in drinking water for 24 weeks (12 weeks pre-implantation + 12 weeks post-implantation); *n* per group: 6	To investigate the effect of long-term daily caffeine intake on the osseointegration process in rats.	Long-term caffeine intake significantly increased implant stability and mineralized bone volume within the implant threads, indicating enhanced long-term osseointegration.	2021 [109]

OPG—osteoprotegerin. RANKL—receptor activator of nuclear factor kappa Β-ligand. TRAP—tartrate-resistant acid phosphatase.

**Table 7:** Effect of caffeine sources (coffee and yerba mate) in experimental in vivo studies.

Animals	Caffeine Dose and Study Conditions	Aim	Effects	Year of Publication/Reference
Male Wistar rats	125 or 275 mg coffee (containing 2.5–5.0% caffeine)/day in the diet for 140 days; *n* per group: 16	To investigate the effect of long-term coffee consumption on bone metabolism in rats.	Chronic consumption of coffee did not change bone turnover markers (serum osteocalcin, bone volume, and number of osteoclasts) in rats.	2001 [110]
Male Wistar rats; tooth extraction performed at postnatal day 60	Prenatal exposure: maternal coffee intake (50 mg/mL infusion, ad libitum) for 30 days before mating and during pregnancy Postnatal exposure: coffee intake (50 mg/mL infusion, ad libitum) from birth to approximately postnatal day 60 End of the experiment 7,14 and 21 days after tooth extraction; *n* per group: 7	To investigate the effect of chronic coffee intake on calcium homeostasis and alveolar bone repair after tooth extraction in growing rats.	Chronic coffee intake increased plasma and urinary calcium levels and decreased bone calcium content, BMD, and bone volume, resulting in delayed bone repair.	2010 [111]
40-day-old Wistar male rats	Coffee: infusion (100 g of coffee powder in 1 L of boiling water) administered for 10 weeks after surgery. Surgery performed 6 weeks after the start of coffee treatment; *n* per group: 5	To investigate the effect of coffee consumption alone and with cigarette smoke inhalation on bone healing and osseous integration of hydroxyapatite implants in rats.	Coffee decreased the formation of new bone and osseous integration. Cigarette smoke inhalation enhanced the unfavorable coffee effect.	2013 [112]
30-day-old female Sprague Dawley rats	Yerba mate: filtered infusion (370 mg/L caffeine; intake 20–70 mg/kg/day) administered for 90 days; animals fed diet containing 0.2% or 0.9% calcium; *n* per group: 6	To investigate the effects of yerba mate infusion on BMD, bone histomorphometry, and biomechanical properties in rats.	Yerba mate consumption did not adversely affect bone parameters in rats. It partially attenuated trabecular bone loss induced by calcium deficiency but did not prevent biomechanical deterioration.	2015 [113]
Male Wistar rats; tooth extraction performed at postnatal day 60	Prenatal exposure: maternal coffee intake (50 mg/mL infusion, ad libitum) for 30 days before mating and during pregnancy Postnatal exposure: coffee intake (50 mg/mL infusion, ad libitum) from birth to approximately postnatal day 60 End of the experiment 7,14 and 21 days after tooth extraction; *n* per group: 5	To investigate the effects of prenatal caffeine exposure and long-term coffee consumption on alveolar bone healing after tooth extraction in growing rats.	Chronic exposure to coffee significantly reduced new bone formation and delayed alveolar bone healing after tooth extraction.	2015 [103]
3-month-old male Wistar rats	Yerba mate: instant powder 20 mg/kg/day i.g. for 28 days before and 28 days after tooth extraction; *n* per group: 8	To investigate the effect of yerba mate on alveolar socket healing after tooth extraction.	Yerba mate increased bone area and osteocyte density in the middle third of the alveolar socket after tooth extraction.	2018 [114]
16-month-old female Wistar rats	Yerba mate: instant powder 20 mg/kg/day by gavage for 28 days; *n* per group: 10	To investigate the effects of yerba mate on bones of old rats.	Yerba mate mitigated bone deterioration in old rats.	2018 [115]
2–3-month-old male Wistar rats with experimental periodontitis	Robusta coffee bean extract: 6.25%, 12.5%, 25%, or 50% rinsed into the periodontal pocket for 7 days; *n* per group: 4	To investigate the effect of Robusta coffee bean extract on antibacterial activity and alveolar bone regeneration in a rat periodontitis model.	Robusta coffee bean extract at a 50% concentration increased osteoblast numbers, decreased osteoclast numbers, and enhanced BMP-2 expression, indicating improved alveolar bone repair in rats with experimentally induced periodontitis.	2023 [116]

BMD—bone mineral density.

**Table 8:** Effect of caffeine on osteoblasts and osteoblast-like cells in vitro.

Model	Caffeine Concentrations/Study Conditions	Aim	Effects	Year of Publication/Reference
Calvarial osteoblast cultures from 16-day-old chicks	Caffeine: 0.1–0.4 mM	To investigate the effects of caffeine on osteoblast growth, differentiation, and the formation and mineralization of the extracellular matrix in vitro.	Caffeine caused a dose-dependent decrease in osteocalcin, ALP, and collagen, with the strongest inhibition before mineral deposition. At 0.4 mM, collagen remained inhibited on day 28, and mineralization was not initiated due to impaired extracellular matrix formation.	1991 [117]
Rat osteoblast-like UMR106-01 cells treated with/without prostaglandin E_2_ (PGE_2_)	Caffeine: 0.1 or 1.0 mM	To investigate the possible interaction between caffeine and PGE_2_ on the proliferation of osteoblast-like cells in vitro.	Caffeine at a concentration of 0.1 mM did not inhibit cell proliferation; however, at 1.0 mM, it significantly reduced the number of cells.	1999 [118]
Human osteoblastic SaOS-2 cells treated with/without dexamethasone	Caffeine: 0.5 mM	To investigate the role of caffeine in modulating glucocorticoid receptor function and glucocorticoid-initiated gene expression in human osteoblastic cells.	Caffeine significantly enhanced dexamethasone-induced reporter gene expression in human osteoblast cells. This effect was further enhanced by forskolin and inhibited by RU486 and was dependent on intracellular cyclic AMP levels.	2005 [119]
Primary osteoblasts derived from newborn Wistar rat calvaria cells	Caffeine: 0.1–100 mM	To investigate the effect of caffeine on osteoblast metabolism and viability in vitro.	Caffeine concentrations ≥1 mM significantly reduced osteoblast viability from day 3. On day 7, a reduction in osteoblast activity was observed at most concentrations. Exposure to 10 mM caffeine significantly reduced the formation of ALP-positive colonies and calcified nodules. Additionally, intracellular levels of ALP, LDH, and PGE_2_ were significantly reduced, while the level of LDH released into the medium was significantly increased.	2006 [120]
Human osteoblast-like cell lines U2-OS and MG-63 treated with 1,25(OH)_2_D_3_	Caffeine: 0.2–10 mM	To investigate the effect of caffeine on VDR protein expression and on 1,25(OH)_2_D_3_-mediated osteoblastic activity in human osteoblast cells.	Caffeine dose dependently decreased 1,25(OH)_2_D_3_-induced protein expression (by ~50% at 1 mM and ~70% at 10 mM). It also reduced 1,25(OH)_2_D_3_-stimulated ALP activity (~50% at 1 mM). Caffeine alone did not affect basal ALP activity in osteoblastic cells.	2007 [121]
Human osteoblast cell line hFOB 1.19 cells	Caffeine: 0.25–2 mM	To investigate the effect of caffeine on cell death mechanisms and signaling pathways associated with survival in human osteoblasts.	Caffeine reduced osteoblast viability in a dose-dependent manner and induced apoptosis (and, to a lesser extent, necrosis). Caffeine also increased ROS levels, elevated the Bax/Bcl-2 ratio, caused loss of mitochondrial membrane potential, and activated caspase-9 and caspase-3. In addition, caffeine induced PAK2 cleavage and JNK activation.	2008 [122]
MC3T3-E1 cells (murine preosteoblastic cells)	Caffeine: 0.001–1 mM	To investigate the effect of caffeine on the expression of RANKL and OPG proteins in mouse osteoblast cells.	Caffeine increased RANKL expression and decreased OPG expression in MC3T3-E1 cells, resulting in an increase in the RANKL/OPG ratio.	2011 [80]
Primary calvarial osteoblasts isolated from neonatal Wistar rats	Caffeine: 25, 50 or 100 mg/kg/day (maternal exposure during pregnancy)	To investigate the effect of prenatal caffeine exposure on the osteogenic potential of osteoblasts isolated from newborn rats.	In osteoblasts isolated from newborn rats born to mothers that received 50 mg/kg/day of caffeine during pregnancy, there was increased ALP activity, collagen synthesis, a higher percentage of mineralization nodules, and increased expression of osteogenic markers, including sialoprotein, Runx-2, ALP, osteocalcin, osteopontin, and type I collagen. Groups receiving doses of 25 and 100 mg/kg/day of caffeine showed changes in the transcription of some osteogenic genes but did not show increased mineralization.	2015 [123]
MC3T3-E1 cells (murine preosteoblastic cells)	Caffeine: 0.66, 1.66, or 3.33 µg/mL	To investigate the effect of caffeine alone or with other yerba mate (rutin, chlorogenic acid) components on the viability of preosteoblastic cells in vitro.	In MC3T3-E1 cells, all concentrations of caffeine significantly increased cell viability. The combinations of rutin and caffeine caused a significant increase in MC3T3-E1 cell viability. Chlorogenic acid and caffeine also significantly increased MC3T3-E1 cell viability.	2024 [124]
MC3T3-E1 cells (murine preosteoblastic cells)	Caffeine: 3.125–50 μg/mL	To investigate the effect of caffeine on preosteoblast differentiation and osteogenesis as well as signaling pathways.	Caffeine at all concentrations significantly increased ALP activity in MC3T3-E1 cells. Caffeine concentrations of 12.5 μg/mL significantly increased Runx2 and Osterix expression and inhibited the phosphorylation of AKT, IκBα, P65, ERK, JNK, and P38. However, 50 μg/mL of caffeine significantly increased the phosphorylation of IκBα, P65, JNK and AKT compared to the group receiving 12.5 μg/mL, leading to disruption of the balance between osteoblastogenesis and osteoclastogenesis.	2024 [96]

AKT—protein kinase B. ALP—alkaline phosphatase. AMP—adenosine monophosphate. Bax—Bcl2-associated X protein. Bcl-2—B-cell lymphoma-2. ERK—extracellular signal-regulated kinases. IκBα—nuclear factor of kappa light polypeptide gene enhancer in B-cell inhibitor alpha JNK—c-Jun N-terminal kinase. LDH—lactate dehydrogenase. OPG—osteoprotegerin. PAK2—p21 activated protein kinase 2. PGE_2_—prostaglandin E_2_. RANKL—receptor activator of nuclear factor kappa-Β ligand. ROS—reactive oxygen species. VDR—vitamin D receptor.

**Table 9:** Effect of caffeine on mesenchymal stem/stromal cells in vitro.

Model	Caffeine Concentrations/Study Conditions	Aim	Effects	Year of Publication/Reference
Rat bone marrow-derived mesenchymal stromal cells (BMSCs)	Caffeine: 0.1–1 mM	To investigate the effect of caffeine on the viability and osteogenic differentiation of BMSCs.	Caffeine (0.1–1 mM) increased intracellular cAMP levels and decreased cell viability in a concentration-dependent manner, inducing necrosis and apoptosis. It also downregulated Cbfa1/Runx2, Col-I and ALP, reduced ALP activity and calcium deposition, and increased osteocalcin mRNA and protein levels during BMSCs osteogenesis.	2010 [125]
Primary rat adipose-derived stem cells (ADSCs)	Caffeine: 0.1–1 mM	To investigate the effect of caffeine on osteogenic differentiation and mineralization of ADSCs.	Caffeine (0.1 mM) significantly enhanced osteogenic differentiation and mineralization and significantly increased the expression of OPG, RUNX2, and SIRT1 in ADSCs. However, 0.3 and 1 mM caffeine significantly inhibited osteogenic differentiation and decreased the levels of OPG, RUNX2, and SIRT1.	2013 [126]
M2-10B4 mouse bone marrow stromal cells	Caffeine: 0.1–1 mM	To investigate the effect of caffeine on osteoblast differentiation in M2-10B4 mouse bone marrow stromal cells by assessing ALP activity and ALP and osteocalcin gene expression.	Caffeine (0.1 mM) significantly increased ALP activity and increased the expression of ALP and osteocalcin genes. However, 0.3 and 1 mM caffeine significantly decreased ALP activity and decreased the expression of ALP and osteocalcin in M2-10B4 cells.	2013 [126]
Bone marrow mesenchymal stem cells (BMSCs)	Caffeine: 25, 50 or 100 mg/kg/day *p.o.* (maternal exposure during pregnancy and lactation)	To investigate the effect of caffeine on the osteogenic differentiation of BMSCs derived from the offspring of rats exposed during pregnancy and lactation.	Prenatal and lactational caffeine exposure significantly inhibited osteogenic differentiation of BMSCs derived from offspring. Caffeine intake caused reduced ALP activity (50–100 mg/kg), decreased expression of osteogenic markers (Runx2, BSP, ALP, Col-I), decreased osteocalcin mRNA levels (50 mg/kg), and reduced mineralization in all treated groups.	2016 [127]
Bone marrow-derived mesenchymal stromal cells (BMSCs) isolated from osteoporotic rats	Caffeine: 0.1–0.4 mM	To investigate the effect of estradiol on the caffeine effect on BMSCs.	Caffeine reduced BMSCs proliferation in a dose-dependent manner and increased apoptosis (minimum inhibitory concentration: 0.2 mM). It also inhibited osteogenic differentiation, reflected by decreased mineralization and reduced expression of osteogenic markers (Runx2, Col-I, osteocalcin) while increasing the RANKL/OPG ratio and intracellular cAMP levels. The effects were blunted by estradiol.	2018 [94]
Bone marrow mesenchymal stem cells (BMSCs) from female C57BL/6J mice	Caffeine: 0.01–1.0 mM	To investigate the effect of caffeine on the biological functions and differentiation potential of BMSCs.	Caffeine significantly reduced the viability, proliferation, migration, and pluripotency of BMSCs and also significantly inhibited osteogenic differentiation, resulting in reduced mineralization and decreased expression of osteogenic markers (Runx2, Osterix, osteocalcin). Caffeine significantly promoted the adipogenic differentiation of BMSCs, causing increased lipid droplet formation and increased expression of adipogenic markers.	2021 [88]
Human dental pulp stem cells (DPSCs)	Caffeine: 0.125–8.03 µM	To investigate the effect of caffeine on DPSCs.	Caffeine at a concentration of 8.03 µM significantly increased the adipogenic differentiation of DPSCs. No statistically significant effect on cell viability, cell proliferation, or COX-1 and COX-2 expression was observed.	2025 [128]

ADSCs—adipose-derived stem cells. ALP—alkaline phosphatase. BMSCs—bone marrow-derived mesenchymal stem cells. cAMP—cyclic adenosine monophosphate. Col-I—collagen I. COX-1—cyclooxygenase 1. COX-2—cyclooxygenase 2. DPSCs—dental pulp stem cells. OPG—osteoprotegerin. *p.o.*—per os (orally). RANKL—receptor activator of nuclear factor kappa-Β ligand.

**Table 10:** Effect of caffeine on osteoclasts in vitro.

Model	Caffeine Concentrations	Aim	Effects	Year of Publication/Reference
Bone marrow cells isolated from femurs of 5-week-old ICR mice induced by RANKL and M-CSF	Caffeine: 0.005 or 0.01 mM	To investigate the effect of caffeine on osteoclast differentiation from bone marrow HSCs.	Caffeine increased osteoclast differentiation from bone marrow cells, demonstrated by an increased number of TRAP-positive multinucleated cells. Caffeine also increased the bone resorption activity of osteoclasts, shown by an increased formation of pits in the resorption assay.	2011 [80]
RAW264.7 murine monocyte/macrophage cell line treated with/without RANKL	Caffeine: 1, 10 or 100 μM	To investigate the effect of caffeine on osteoclast differentiation and maturation and to determine the associated molecular mechanisms.	In RAW264.7 cells, caffeine increased osteoclast differentiation and maturation, probably through p38 MAPK activation.	2013 [48]
RAW264.7 murine monocyte/macrophage cell line treated with RANKL	Caffeine: 3.125, 12.5, or 50 μg/mL	To investigate the effect of caffeine on osteoclast differentiation and underlying molecular mechanisms in RANKL-induced RAW264.7 cells.	Lower caffeine concentrations inhibited RANKL-induced osteoclastogenesis in RAW264.7 cells. Also, the phosphorylation of MAPK and NF-κB signaling proteins was inhibited. High caffeine concentrations increased the number of TRAP-positive multinucleated osteoclasts.	2024 [96]
RAW264.7 murine monocyte/macrophage cell line treated with RANKL	Caffeine: 1–300 μM	To investigate the effects of caffeine on osteoclastogenesis and bone resorption activity in RANKL-induced RAW264.7 cells.	Low concentrations (1–10 μM) of caffeine promoted RANKL-induced osteoclast differentiation and bone resorption activity in RAW264.7 cells (increased numbers of TRAP-positive multinucleated osteoclasts and increased release of fluorescent resorption markers). However, higher concentrations of caffeine (30–300 µM) significantly inhibited osteoclast differentiation and bone resorption activity. Moreover, A_1_R inhibition reduced the stimulatory effect of caffeine on osteoclast formation.	2025 [130]

A_1_R—adenosine A_1_ receptor. HSCs—hematopoietic stem cells. MAPK—mitogen-activated protein kinase. M-CSF—macrophage colony-stimulating factor. NF-κB—nuclear factor kappa-light-chain-enhancer of activated B cells. RANKL—receptor activator of nuclear factor kappa-Β ligand. TRAP—tartrate-resistant acid phosphatase.

**Table 11:** Effect of caffeine on other bone-related models in vitro.

Model	Caffeine Concentrations	Aim	Effects	Year of Publication/Reference
Neonatal calvaria isolated from 6-day-old Swiss-Webster, Vancouver mice	Caffeine: 0.026–2.6 mM	To investigate the effect of caffeine on bone calcium release from neonatal mouse calvaria in an in vitro culture system.	Caffeine did not significantly affect calcium release into the culture medium and did not cause morphological changes in the calvarial bone.	1988 [131]
Neonatal mouse calvaria	Caffeine: 0.1–1 mM	To investigate the effect of caffeine on bone resorption (^45^Ca release) in vitro in organ culture of neonatal mouse calvaria.	Caffeine (0.3 or 1 mM) increased the release of ^45^Ca from the calvaria of newborn mice after 120 h of culture, indicating stimulation of bone resorption, while no effect was observed after 48 h. The effect was concentration-dependent, with stimulation observed at caffeine concentrations of 0.3 and 1 mM.	1992 [132]
Neonatal mouse calvaria (6-day-old ICR mice)	Caffeine: 0.005–0.1 mM	To investigate the effect of caffeine on PGE_2_ production and inflammatory signaling involved in osteoclast-related bone metabolism in calvarial bone tissue.	Caffeine increased COX-2 protein expression and PGE_2_ production in neonatal mouse calvarial bone tissue culture.	2011 [80]
Human periodontal ligament cells (PDL)	Caffeine: 0.01 mM	To investigate the effect of caffeine on PDL cells under mechanical compression.	Caffeine (0.01 mM) significantly increased compression-induced expression of RANKL, COX-2, and PGE_2_ production in PDL cells. The culture medium from PDL cells under compression significantly increased formation of TRAP-positive osteoclasts in the co-culture of osteoblasts/osteoclast precursors.	2016 [104]
Primary growth plate chondrocytes of 17-day-old rats	Caffeine: 0.1–1 mM	To investigate the effect of caffeine on the proliferation and chondrogenic differentiation of growth plate chondrocytes.	Caffeine significantly decreased proliferation of primary rat growth plate chondrocytes. Caffeine also reduced extracellular matrix production and mineralization and decreased ALP activity. Additionally, caffeine decreased expression of the chondrogenic markers Aggrecan, type II collagen, and type X collagen.	2017 [83]
MLO-Y4 osteocyte-like cells	Caffeine: 0.66, 1.66, or 3.33 µg/mL	To investigate the effect of caffeine alone or in combination with other yerba mate components on osteocyte viability in vitro.	Caffeine significantly increased the viability of MLO-Y4 osteocytes at the two highest concentrations. Some combinations of caffeine and rutin also increased osteocyte viability. However, the combination of caffeine and chlorogenic acid had no effect on the viability of MLO-Y4 cells.	2024 [124]

ALP—alkaline phosphatase. COX-2—cyclooxygenase 2. PDL—periodontal ligament cells. PGE_2—_prostaglandin E_2_. RANKL—receptor activator of nuclear factor kappa-Β ligand. TRAP—tartrate-resistant acid phosphatase.

## Data Availability

No new data were generated for this study.
